# Prediction of postoperative cardiopulmonary complications after lung resection in a Chinese population: A machine learning-based study

**DOI:** 10.3389/fonc.2022.1003722

**Published:** 2022-09-23

**Authors:** Guanghua Huang, Lei Liu, Luyi Wang, Shanqing Li

**Affiliations:** Department of Thoracic Surgery, Peking Union Medical College Hospital, Chinese Academy of Medical Sciences & Peking Union Medical College, Beijing, China

**Keywords:** lung cancer, prediction model, machine learning, postoperative complication, ppoFEV1%, FEV1/FVC

## Abstract

**Background:**

Approximately 20% of patients with lung cancer would experience postoperative cardiopulmonary complications after anatomic lung resection. Current prediction models for postoperative complications were not suitable for Chinese patients. This study aimed to develop and validate novel prediction models based on machine learning algorithms in a Chinese population.

**Methods:**

Patients with lung cancer receiving anatomic lung resection and no neoadjuvant therapies from September 1, 2018 to August 31, 2019 were enrolled. The dataset was split into two cohorts at a 7:3 ratio. The logistic regression, random forest, and extreme gradient boosting were applied to construct models in the derivation cohort with 5-fold cross validation. The validation cohort accessed the model performance. The area under the curves measured the model discrimination, while the Spiegelhalter z test evaluated the model calibration.

**Results:**

A total of 1085 patients were included, and 760 were assigned to the derivation cohort. 8.4% and 8.0% of patients experienced postoperative cardiopulmonary complications in the two cohorts. All baseline characteristics were balanced. The values of the area under the curve were 0.728, 0.721, and 0.767 for the logistic, random forest and extreme gradient boosting models, respectively. No significant differences existed among them. They all showed good calibration (p > 0.05). The logistic model consisted of male, arrhythmia, cerebrovascular disease, the percentage of predicted postoperative forced expiratory volume in one second, and the ratio of forced expiratory volume in one second to forced vital capacity. The last two variables, the percentage of forced vital capacity and age ranked in the top five important variables for novel machine learning models. A nomogram was plotted for the logistic model.

**Conclusion:**

Three models were developed and validated for predicting postoperative cardiopulmonary complications among Chinese patients with lung cancer. They all exerted good discrimination and calibration. The percentage of predicted postoperative forced expiratory volume in one second and the ratio of forced expiratory volume in one second to forced vital capacity might be the most important variables. Further validation in different scenarios is still warranted.

## Introduction

Lung cancer is the most common cancer in China, accounting for 23.9% of new cancer cases and 18.1% of cancer deaths (1). Surgery is a mainstay in the treatment of lung cancer. Postoperative cardiopulmonary complications may occur in approximately 20% of patients (2, 3). They are associated with higher risks of readmission, chronicity of these complications, and cancer recurrence (2–5). Therefore, reducing the incidence of complications is of vital importance for clinicians. Some strategies have been developed, such as prehabilitation and standardized enhanced recovery after surgery programs (6, 7). Among them, preoperational screening has the highest cost-effectiveness. An accurate prediction model is critical for screening and can enhance shared preoperative decision-making and medical care quality monitoring.

Several prediction models have been established over the past few years, including the Brunelli, Eurolung, and parsimonious Eurolung models (8–10). Some comorbidity risk calculators, such as the age-adjusted Charlson Comorbidity Index (ACCI), have also shown potential predictive efficacy (11). Most of these models were built based on the European population. However, these models did not perform satisfactory discrimination among the Chinese population due to patient characteristics discrepancies, with the values of the area under the curve (AUC) less than 0.7 (12). Predictive studies based on Chinese populations have mainly focused on a certain type of complication, a subgroup of patients, or a particular predictor (13–18). For example, Li et al. developed two prediction models for pneumonia and arrhythmia, but they did not pay attention to prolonged air leak, atelectasis, and other complications (16). Currently, no generalized models have been established, which could predict the overall incidence of complications and be suitable for the broad Chinese population. Recent advances in machine learning enhance the development of prediction models. The random forest and extreme gradient boosting (XGBoost) algorithms show promising performance and often outperform the logistic model (19). However, only a few studies applied machine learning to develop models for postoperative cardiopulmonary complications (20).

Therefore, this study aimed to develop and validate generalized prediction models for postoperative cardiopulmonary complications based on a Chinese population. It would be the first to address the needs of Chinese patients while applying machine learning. This article was presented based on the transparent reporting of a multivariable prediction model for individual prognosis or diagnosis reporting checklist (21).

## Materials and methods

### Patient selection

This retrospective study collected information on patients who underwent lung surgeries at our center from September 1, 2018 to August 31, 2019. Patients were eligible if they were > 18 years old, had undergone anatomic lung surgeries, and had no prior neoadjuvant therapies. Patients who lacked lung function metrics or were confirmed to have non-lung cancer by pathological reports were excluded. The study protocol was reviewed and approved by the Institutional Review Board of Peking Union Medical College Hospital (No. K2038). The requirement for informed consent was waived due to the study’s retrospective nature.

### Variables and outcomes

Information on sex, age, body mass index (BMI), history of smoking and alcohol intake, comorbidities, forced expiratory volume in one second (FEV1), forced vital capacity (FVC), surgical procedure, and extended resection was collected. The Charlson Comorbidity Indices (CCI) and FEV1/FVC were calculated. The percentage of predicted postoperative forced expiratory volume in one second (ppoFEV1%) was calculated as follows: (FEV1/predicted FEV1) x (1-a/b), where a was the number of removed segments and b was the number of total segments (22). The predicted FEV1 and FVC were estimated using formulas initially developed from Chinese populations rather than Caucasian populations (23).

Outcome variables included prolonged air leakage, pneumonia, pulmonary edema, atelectasis, arrhythmia, acute myocardial infarction, and other complications listed at length in our previous study (12). Their definitions were based on the instructions of the Society of Thoracic Surgeons and the European Society of Thoracic Surgeons (24).

### Model development and validation

Pearson or Spearman correlation analysis was first performed using the ‘stats’ R package for age, ACCI, CCI, and lung function metrics, according to their normality. Those pairs with correlation coefficients > 0.7 were carefully screened based on clinical experiences. The remaining variables were used to develop predictive models through three algorithms: logistic regression, random forest, and XGBoost. The entire dataset was randomly split into a derivation cohort and a validation cohort at a 7:3 ratio using the ‘caret’ R package. As for the logistic model performed using the ‘stats’ R package, all variables were screened by univariate analysis first, and then those with p values < 0.05 were further underwent backward stepwise selection. Akaike’s information criterion was implemented during stepwise selection, simplifying the model and maintaining its efficacy. A nomogram was plotted using the ‘rms’ package based on the logistic model to facilitate model interpretation. Random forest, performed with the ‘randomForest’ package, is a bagging-based machine learning method that reduces the risk of overfitting, determines feature importance, and has high flexibility. XGBoost, performed using the ‘xgboost’ package, is a boosting-based method designed to be highly efficient, flexible, and portable. Random searches and 5-fold cross-validation performed with the ‘mlr’ package were employed for hyperparameter tuning of the machine learning models. The random forest and XGBoost models were interpreted based on the mean decreased Gini index and total gain, respectively.

The models were constructed and internally validated in the derivation cohort. Five-fold cross-validation is a well-accepted method for internal validations. The validation cohort was used solely to measure the model performance. Model discrimination and calibration must be reported, whereas sensitivity, specificity, and accuracy are optional. The AUC assessed discrimination. An AUC > 0.7 was regarded as good discrimination, while AUC > 0.6 meant acceptable discrimination. DeLong’s test was used to compare the differences between two AUCs. Calibration curves were plotted after correcting for bias using 1000 bootstrap iterations. A perfect curve is closely fitted to the diagonal line. In addition, the Spiegelhalter z test assessed the calibration accuracy, and a non-significant p value indicated good calibration (25, 26). The AUC and DeLong’s test were performed with the ‘pROC’ package, while calibration curves and z test were performed with the ‘rms’ package. Numeric variables with normal distribution were described as means and standard deviations, and non-normally distributed variables were expressed as medians and interquartile ranges. Categorical variables were presented as counts and percentages. Group differences of numeric variables were tested using the t-test or the median test, and those of categorical variables were compared using the chi-square test or Fisher’s exact test, according to their distributions. Statistical significance was set at p < 0.05. All analyses were performed using R version 4.1.2 (RRID: SCR_001905).

## Results

**Figure 1:**
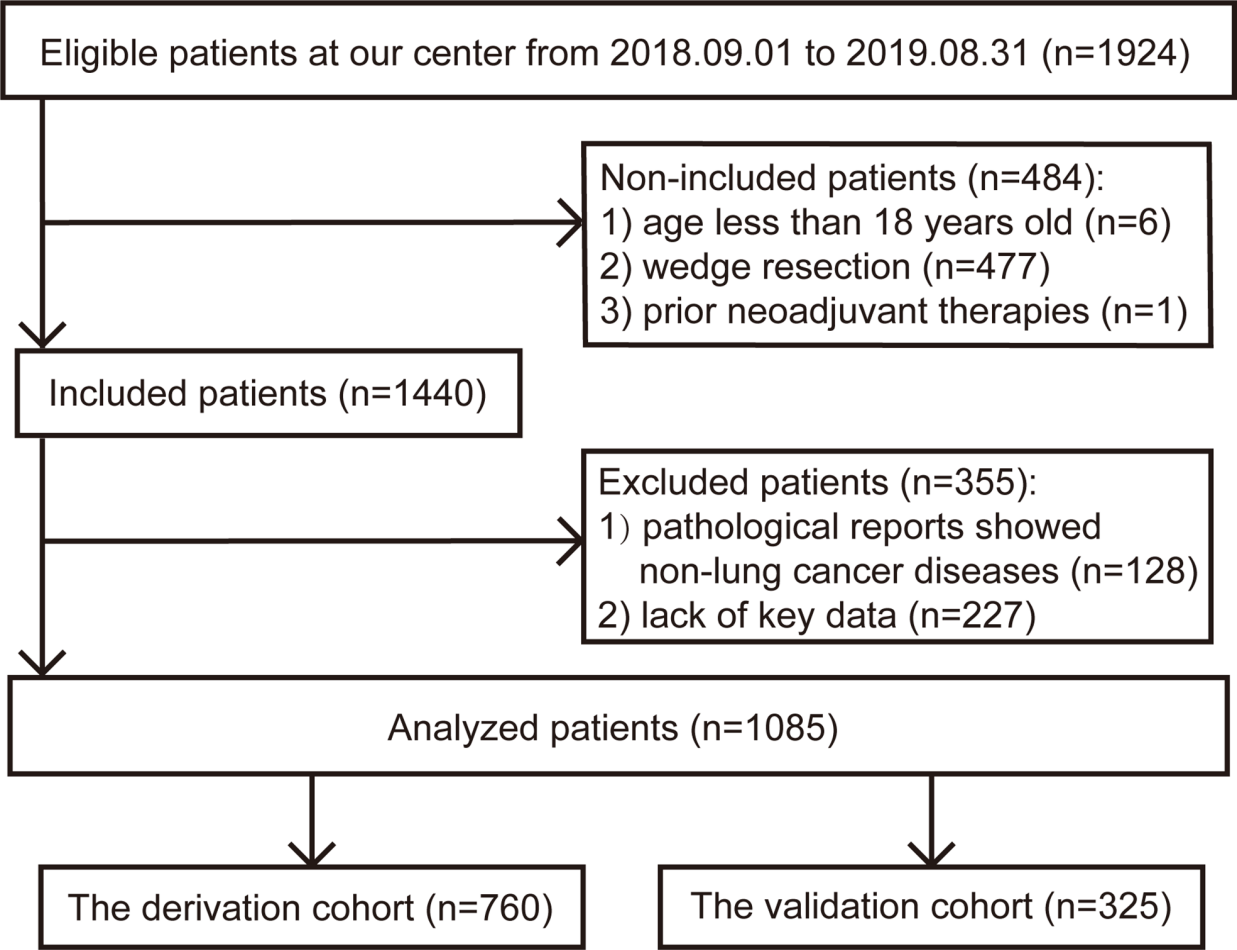
The flow chart of patient selection.

The flow chart of patient selection is presented in **Figure 1**. After selection, 1085 patients were included in the final analysis, of whom 760 patients (70%) were randomly assigned to the derivation cohort. This was a complete case analysis. No missing data needed to be handled. The correlation coefficients among the aforementioned variables are listed in **Supplementary Table 1**. CCI not ACCI, ppoFEV1% not FEV1 or FEV1%, FVC% not FVC, and FEV1/FVC were selected. The demographic, clinical, and surgical characteristics of the two cohorts are summarized in **Table 1**. All baseline characteristics were balanced. The ppoFEV1%, FVC%, and FEV1/FVC for both cohorts were 76.8 vs. 75.2 (p = 0.094), 89.8 vs. 88.5 (p = 0.192), and 76.0 vs. 75.9 (p = 0.865), respectively. Sixty-four (8.4%) and 26 (8.0%) patients experienced postoperative cardiopulmonary complications in the derivation and validation cohorts, respectively. Details of postoperative cardiopulmonary complications were described in our previous study (12).

**Table 1:** Characteristics of the derivation cohort and the validation cohort.

Terms	Total (N = 1085)	Derivation (N = 760)	Validation(N = 325)	p
Male, N (%)	423 (39.0)	285 (37.5)	138 (42.5)	0.142
Age, mean (SD)	58.4 (10.4)	58.0 (10.6)	59.2 (9.8)	0.100
Body mass index, mean (SD)	24.2 (3.1)	24.2 (3.0)	24.3 (3.3)	0.647
Smoker, N (%)	235 (21.7)	167 (22.0)	68 (20.9)	0.761
Alcohol use, N (%)	139 (12.8)	94 (12.4)	45 (13.8)	0.570
Hypertension, N (%)	312 (28.8)	209 (27.5)	103 (31.7)	0.185
Diabetes mellitus, N (%)	130 (12.0)	92 (12.1)	38 (11.7)	0.928
COPD, N (%)	41 (3.8)	31 (4.1)	10 (3.1)	0.536
Arrhythmia, N (%)	23 (2.1)	16 (2.1)	7 (2.2)	1.000
Coronary artery disease, N (%)	59 (5.4)	39 (5.1)	20 (6.2)	0.593
Cerebrovascular disease, N (%)	23 (2.1)	18 (2.4)	5 (1.5)	0.523
Chronic kidney disease, N (%)	5 (0.5)	4 (0.5)	1 (0.3)	1.000
Interstitial lung disease, N (%)	4 (0.4)	3 (0.4)	1 (0.3)	1.000
CCI, median [IQR]	0 [0-0]	0 [0-0]	0 [0-0]	0.828
ppoFEV1%, mean (SD)	76.3 (14.1)	76.8 (13.8)	75.2 (14.8)	0.094
FVC%, mean (SD)	89.4 (14.0)	89.8 (13.9)	88.5 (14.2)	0.192
FEV1/FVC, mean (SD)	76.0 (7.9)	76.0 (7.8)	75.9 (8.2)	0.865
Thoracotomy, N (%)	25 (2.3)	18 (2.4)	7 (2.2)	1.000
Surgical procedures, N (%)				0.774
Segmentectomy	209 (19.3)	146 (19.2)	63 (19.4)	
Lobectomy	863 (79.5)	604 (79.5)	259 (79.7)	
Bilobectomy	11 (1.0)	9 (1.2)	2 (0.6)	
Pneumonectomy	2 (0.2)	1 (0.1)	1 (0.3)	
Extended resection, N (%)	12 (1.1)	10 (1.3)	2 (0.6)	0.488
Postoperative complications, N (%)	90 (8.3)	64 (8.4)	26 (8.0)	0.912

OR, odds ratio; CI, confidence interval; SD, standard deviation; COPD, chronic obstructive pulmonary disease; CCI, the Charlson Comorbidity Index; IQR, interquartile range; ppoFEV1%, the percentage of predicted postoperative forced expiratory volume in one second; FVC%, the percentage of forced vital capacity; FEV1/FVC, the ratio of forced expiratory volume in one second to forced vital capacity.

### Logistic model

**Figure 2:**
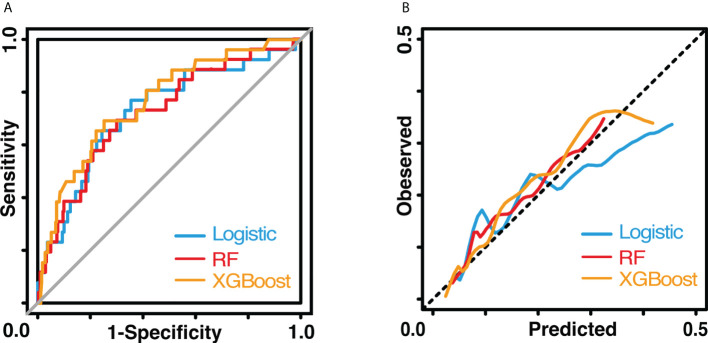
Performance of three models. **(A)** shows the receiver operating characteristic curves. **(B)** shows the calibration curves. The blue line indicates the logistic model The red line indicates the random forest model. The yellow line indicates the XGBoost model.

**Figure 3:**
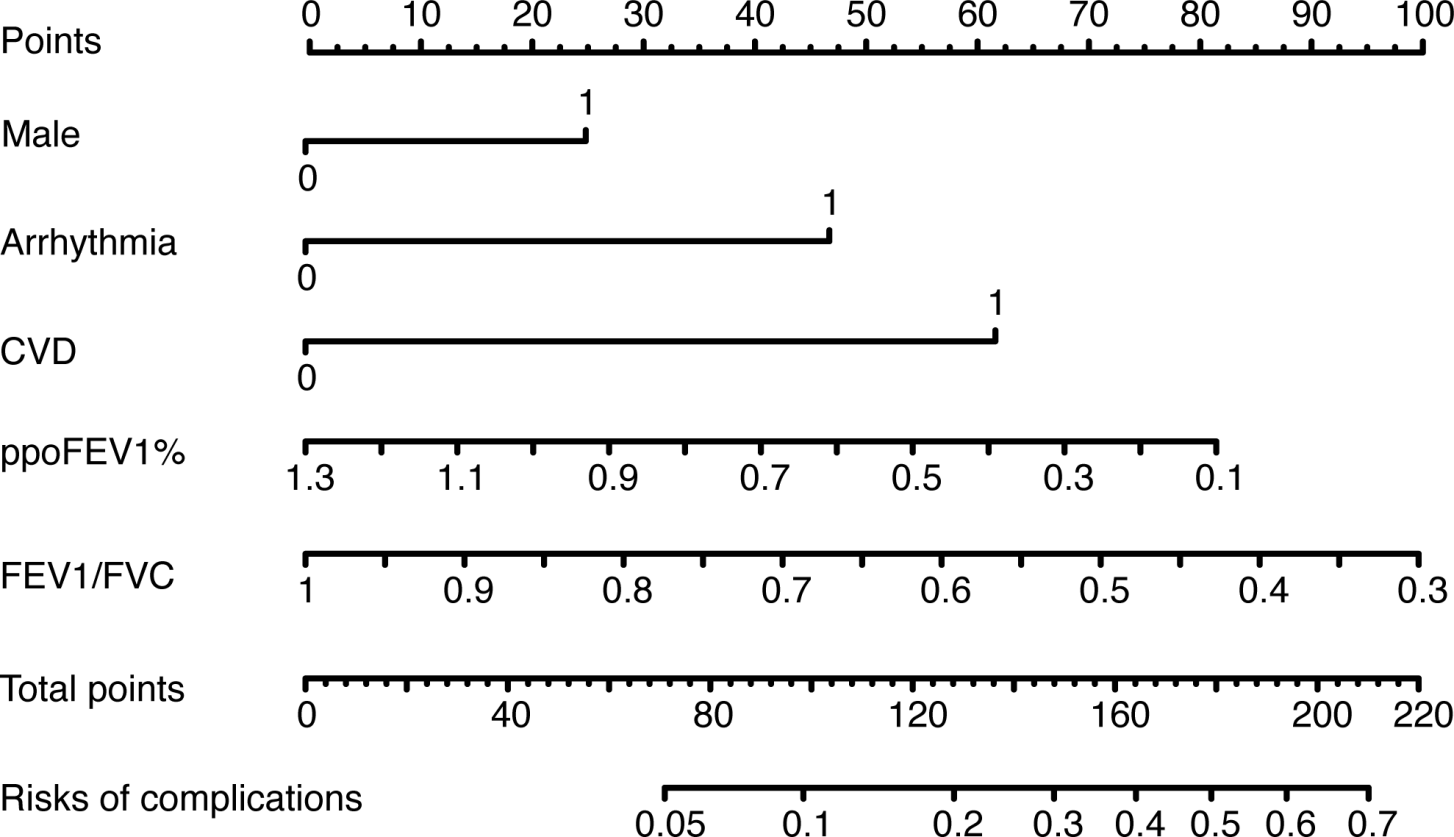
The nomogram of the logistic model. CVD, cerebrovascular disease; ppoFEV1%, the percentage of predicted postoperative forced expiratory volume in one second; FEV1/FVC, the ratio of forced expiratory volume in one second to forced vital capacity.

The results of the univariate logistic analysis are summarized in **Supplementary Table 2**. Nine variables were statistically significant: male sex, age, smoking status, alcohol use, chronic obstructive pulmonary disease, arrhythmia, cerebrovascular disease, ppoFEV1%, and FEV1/FVC ratio. They were further screened using a backward stepwise selection, which yielded five variables. The coefficients and odds ratios (OR) are listed in **Table 2**. Male sex (OR 1.986, 95% confidence interval [CI] 1.142-3.454, p = 0.015), arrhythmia (OR 3.606, 95%CI 1.095-11.880, p = 0.035), and cerebrovascular disease (OR 5.415, 95%CI 1.852-15.832, p = 0.002) were independent risk factors, while FEV1/FVC (OR 0.020, 95%CI 0.001-0.810, p = 0.038) was an independent protective factor. The logistic equation was as follows: 1.430 + 0.686 × male (yes = 1) + 1.283 × arrhythmia (yes = 1) + 1.689 × cerebrovascular disease (yes = 1) - 1.859 × ppoFEV1% - 3.894 × FEV1/FVC. The mean value of the AUC in the 5-fold cross-validation was 0.722, and its mean accuracy reached 0.787, indicating a qualified model performance for further validation. **Table 3** summarizes the metrics of model performance. The validation cohort also displayed good discrimination (AUC 0.728, 95% CI 0.619-0.836, **Figure 2**) and good calibration (p = 0.656 > 0.05). A nomogram was also plotted to facilitate clinical use **(** **Figure 3** **)**. Moreover, an online calculator of the nomogram can be found at https://onlinepresentation.shinyapps.io/complication.

**Table 2:** Risk factors and their parameters of the logistic model.

Variables	Coefficients	OR (95%CI)	p
Intercept	1.430	–	–
Male	0.686	1.986 (1.142-3.454)	0.015
Arrhythmia	1.283	3.606 (1.095-11.880)	0.035
Cerebrovascular disease	1.689	5.415 (1.852-15.832)	0.002
ppoFEV1%	-1.859	0.156 (0.016-1.543)	0.112
FEV1/FVC	-3.894	0.020 (0.001-0.810)	0.038

OR, odds ratio; CI, confidence interval; ppoFEV1%, the percentage of predicted postoperative forced expiratory volume in one second; FEV1/FVC, the ratio of forced expiratory volume in one second to forced vital capacity.

**Table 3:** Model performance of the logistic model, random forest model and XGBoost model.

	Logistic	Random forest	XGBoost
AUC	0.728	0.721	0.767
95% CI	0.619-0.836	0.614-0.828	0.671-0.862
Spiegelhalter z test	0.656	0.628	0.368
Sensitivity	0.769	0.692	0.692
Specificity	0.645	0.699	0.749
Positive predictive value	0.159	0.167	0.194
Negative predictive value	0.970	0.963	0.966
Accuracy	0.655	0.698	0.745

AUC, area under the curve; CI, confidence interval.

### Machine learning models

**Figure 4:**
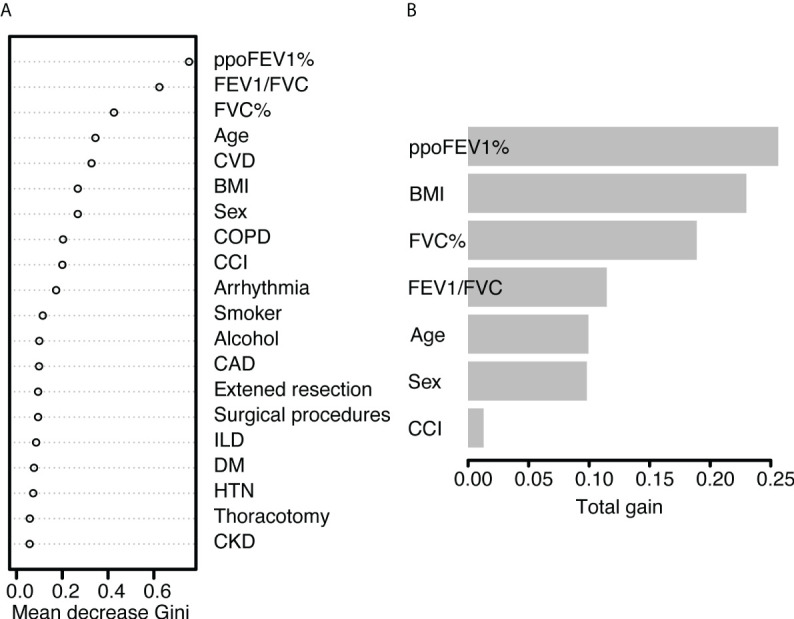
The feature importance of **(A)** the random forest model and **(B)** the XGBoost model. PpoFEV1%, the percentage of predicted postoperative forced expiratory volume in one second; FEV1/FVC, the ratio of forced expiratory volume in one second to forced vital capacity; FVC%, the percentage of forced vital capacity; CVD, cerebrovascular disease; BMI, body mass index; COPD, chronic obstructive pulmonary disease; CCI, the Charlson Comorbidity Index; CAD, coronary artery disease; ILD, Interstitial lung disease; DM, diabetes mellitus; HTN, hypertension; CKD, chronic kidney disease.

In the random forest model, the hyperparameters were set as follows: number of trees = 300, node size = 8, maximum nodes = 8, and mtry = 1. The mean AUC was 0.718 in the 5-fold internal cross-validation. In the validation cohort, the AUC reached 0.721 (95% CI 0.614-0.828), and good calibration was obtained (p = 0.628 > 0.05). The calibration curve of the random forest model was the closest to the diagonal line (**Figure 2**). Sensitivity and specificity were 0.692 and 0.699, respectively. The feature importance is illustrated in **Figure 4A**. PpoFEV1%, FEV1/FVC, FVC%, age, and cerebrovascular disease were the top five important variables. Male sex ranked seventh, while arrhythmia ranked tenth. The mean decreases in the Gini indices of ppoFEV1% and FEV1/FVC were visually higher than others, indicating their prominent roles in prediction.

In the XGBoost model, the tuned hyperparameters are indicated as follows: booster = gbtree; objective = binary:logistic, nround = 97, max_depth = 13, eta = 0.29, min_child_weight = 15.7, subsample = 0.558, colsample_bytree = 0.659, gamma = 0. It performed well in internal validation, with a mean AUC of 0.727. The XGBoost model also showed good discrimination (AUC 0.767, 95% CI 0.671-0.862) and calibration (p = 0.368 > 0.05) simultaneously in the validation cohort. The sensitivity, specificity, and accuracy were 0.692, 0.749, and 0.745, respectively (**Table 3**). **Figure 4B** shows the importance of variables. The top five variables were ppoFEV1%, BMI, FVC%, FEV1/FVC, and age, while male sex ranked sixth.

Although the AUC of the XGBoost model was the highest among the three models, no significant differences were observed after examining by DeLong’s test (logistic vs. random forest, p = 0.801; logistic vs. XGBoost, p = 0.600; random forest vs. XGBoost, p = 0.534).

## Discussion

This was the first study that met Chinese patients’ demand of prediction of postoperative cardiopulmonary complications after lung resection. Three models using various algorithms were developed and validated internally, and all of them showed good discrimination and calibration. PpoFEV1% and FEV1/FVC were identified as the most important predictive factors.

Three algorithms were used for model development: logistic regression, random forest, and XGBoost. They all have their strengths and weaknesses. The conventional logistic regression is strong in convenient implementation, clear presentation, and intuitive interpretation, but weak in capturing nonlinear relationships between outcomes and variables. As for novel machine learning algorithms like random forest and XGBoost, although they are difficult to interpret and apply in clinical settings due to technical problems, they can provide more insights when dealing with high-dimensional and nonlinear datasets. Hence, they are widely applied in prediction using radiomics, genomics, and large databases (27–29). Many studies showed the predictive ability of novel machine learning models was better than that of the logistic model, but Choi et al. still suggested the logistic model should serve as a benchmark due to its easy interpretation (19, 30). Our results showed that the AUC of the logistic model (AUC = 0.728) was lower than that of XGBoost model (AUC = 0.767), but higher than that of random forest model (AUC = 0.721). The p values for DeLong’s test and z test were all greater than 0.05. Therefore, we concluded that the logistic model had non-inferior performance to the random forest and XGBoost models. The possible reason was that the nonlinear relationships were weak, concealing the performance gaps among them. After a thorough consideration of model performance and interpretation, we recommend using the logistic model rather than the random forest or XGBoost model in clinical practice.

The three models had several important variables in common: ppoFEV1%, FEV1/FVC, FVC%, age, and cerebrovascular diseases. These contributed to postoperative cardiopulmonary complications in different ways. PpoFEV1%, FEV1/FVC, and FVC% reflect lung function. Patients’ lung function decreases significantly after surgery. The resistance of small airways increases, while the mucociliary clearance ability decreases. Afterward airway obstruction occurs, resulting in atelectasis, pneumonia, and more complications. On the other hand, the reduced pulmonary perfusion and increased circulatory resistance lead to elevated cardiac load and decreased oxygen supply, causing hypoxemia, arrhythmia, and others. PpoFEV1% is regarded as one of the most important indicators of postoperative cardiopulmonary complications and mortality. One reason is that ppoFEV1% is a synthetic parameter adjusted by height, age, sex, and the extent of surgical resections. Hence, it was also included in the Brunelli, Eurolung model, and European Society Objective Scores (8, 9, 13, 31, 32). A lower ppoFEV1% reflects the reduction of lung volume and decreased lung function. This study showed that a lower FEV1/FVC value, indicating weaker lung function, was associated with a higher risk of complications, which was consistent with results of previous studies (33, 34). FVC%, an adjusted ventilatory function indicator, also served as a predictor in the Brunelli model (8). Old age has been identified as a risk factor for postoperative complications consistently (32, 35–37). Elder patients have poor physical performance in terms of respiratory muscle strength and eliminating pathogens. Cerebrovascular diseases may impair a patient’s neurological function and mobility. Therefore, they are not conducive to postoperative recovery, but are prone to develop complications.

An accurate prediction model shows great importance in multiple settings. Regarding preoperative decision-making, the risk of complications can be easily calculated using the logistic model or nomogram, which makes precise management possible. For example, according to our logistic model, the probability of developing complications would be 36.5% for a male patient with a ppoFEV1% of 60%, an FEV1/FVC of 40%, and no arrhythmia or cerebrovascular diseases. The patient could directly know his risk of complications before surgery, which may help him better weigh the risks against the benefits and would improve compliance during subsequent treatment. Moreover, to gain better outcomes, clinicians could advise the patient to use bronchodilators and perform preoperative pulmonary rehabilitation which significantly improves FVC%, FEV1%, and FEV1/FVC (38–40). Clinicians could also consider performing a segmentectomy rather than lobectomy to improve ppoFEV1% if possible. For hospital managers and policymakers, a risk-adjusted model facilitates medical care quality monitoring. For instance, Pompili et al. used the Eurolung models to evaluate the performance of three thoracic medical centers (41). The rationale for this is clear; if the observed morbidity or mortality are lower than the predicted values, it indicates good performance. Risk models could also help audit the performance of a surgeon, a new instrument, or a novel surgical technique. By collecting these quantitative data, managers and policymakers can further identify root causes of problems and take appropriate actions to improve the quality of care. As regards medical education, a good prediction model helps students recognize the most meaningful factors and perform patient assessments quickly.

Nevertheless, our study has several limitations. First, an inevitable selection bias may exist because of the study’s retrospective nature. For instance, very few patients had chronic kidney disease or interstitial lung disease at our center. Therefore, our results cannot be directly applied to medical centers with different patient distributions. Second, some potentially essential variables could not be captured, which is a common phenomenon in model development using large databases. For example, the percent of diffusion capacity for carbon monoxide of the lung (DLco%) and the maximal oxygen consumption (VO_2_max), which were strongly associated with postoperative complications, were not included in this study (42, 43). DLco% and VO_2_max are not routinely evaluated in many medical centers including ours. Third, the models did not undergo extensive external validation; therefore, their efficacy must be further verified. However, we applied a 5-fold cross-validation for internal validation and tested their performance in a separate cohort, and the models showed consistently good predictive ability.

In conclusion, three models using logistic regression, random forest and XGBoost were developed and validated successfully for the prediction of postoperative cardiopulmonary complications after anatomic lung resection. The models were suitable for Chinese patients. PpoFEV1% and FEV1/FVC may be the most important predictive factors. Extensive external validation is warranted to verify the model’s performance in various clinical scenarios.

## Data availability statement

The raw data supporting the conclusions of this article will be made available by the authors, without undue reservation.

## Ethics statement

The studies involving human participants were reviewed and approved by the Institutional Review Board of Peking Union Medical College Hospital. The ethics committee waived the requirement of written informed consent for participation.

## Author contributions

GH, LL, and SL contributed to conception and design of the study. GH and LL organized the database. GH performed the statistical analysis. GH wrote the first draft of the manuscript. GH and LW wrote sections of the manuscript. All authors contributed to manuscript revision, read, and approved the submitted version

## Funding

This study was funded by the College Student Innovation Training Program of Peking Union Medical College, Beijing (No. 2022zglc06050 to GH).

## Acknowledgments

We would like to thank Editage (www.editage.cn) for English language editing.

## Conflict of interest

The authors declare that the research was conducted in the absence of any commercial or financial relationships that could be construed as a potential conflict of interest.

## Publisher’s note

All claims expressed in this article are solely those of the authors and do not necessarily represent those of their affiliated organizations, or those of the publisher, the editors and the reviewers. Any product that may be evaluated in this article, or claim that may be made by its manufacturer, is not guaranteed or endorsed by the publisher.
