# Supplementary material for: Prediction of postoperative cardiopulmonary complications after lung resection in a Chinese population: A machine learning-based study
Source: Front Oncol. 2022 Sep 23;12:1003722. doi: 10.3389/fonc.2022.1003722 (PMC9539671; doi:10.3389/fonc.2022.1003722)
Supplement: Supplementary file 1 [file DataSheet_1.docx]

Supplementary Material

**Supplementary Table 1.** Correlation coefficients among variables

| Variable 1 | Variable 2 | Coefficient |
| --- | --- | --- |
| Age | ACCI | **0.884** |
| Age | CCI | 0.231 |
| FEV1 | FEV1% | 0.613 |
| FEV1 | ppoFEV1% | 0.541 |
| FEV1 | FEV1/FVC | 0.317 |
| FEV1 | FVC | **0.907** |
| FEV1 | FVC% | **0.727** |
| ppoFEV1% | FEV1% | **0.885** |
| ppoFEV1% | FVC | 0.374 |
| ppoFEV1% | FVC% | 0.572 |
| ppoFEV1% | FEV1/FVC | 0.453 |
| FVC% | FVC | **0.829** |
| FVC% | FEV1% | 0.661 |
| FVC% | FEV1/FVC | -0.136 |
| FEV1/FVC | FEV1% | 0.517 |
| FEV1/FVC | FVC | -0.096 |
| FVC | FEV1% | 0.421 |

FEV1, forced expiratory volume in one second; ppoFEV1%, the percentage of predicted postoperative forced expiratory volume in one second; FVC, forced vital capacity; FEV1/FVC, the ratio of forced expiratory volume in one second to forced vital capacity.

**Supplementary Table 2.** Results of the univariate analysis

| Variables | OR (95CI%) | p |
| --- | --- | --- |
| Male | 2.657 (1.576-4.480) | **<0.001** |
| Age | 1.029 (1.003-1.057) | **0.030** |
| Body mass index | 0.979 (0.897-1.067) | 0.627 |
| Smoker | 1.840 (1.059-3.198) | **0.031** |
| Alcohol use | 1.935 (1.009-3.713) | **0.047** |
| Hypertension | 1.320 (0.763-2.283) | 0.321 |
| Diabetes mellitus | 1.576 (0.791-3.140) | 0.196 |
| Chronic obstructive pulmonary disease | 3.439 (1.420-8.326) | **0.006** |
| Arrhythmia | 3.800 (1.189-12.145) | **0.024** |
| Interstitial lung disease | 5.508 (0.493-61.588) | 0.166 |
| Chronic kidney disease | 3.667 (0.376-35.770) | 0.264 |
| Cerebrovascular disease | 5.897 (2.135-16.287) | **0.001** |
| Coronary artery disease | 2.078 (0.836-5.165) | 0.115 |
| Charlson Comorbidity Index | 1.205 (0.931-1.560) | 0.157 |
| Thoracotomy | 1.371 (0.308-6.100) | 0.679 |
| Types of surgeries | 1.408 (0.740-2.676) | 0.297 |
| Segmentectomy | Reference | Reference |
| Lobectomy | 1.281 (0.635-2.586) | 0.489 |
| Bilobectomy | 3.886 (0.712-21.220) | 0.117 |
| Pneumonectomy | 0.000 (0.000-Inf) | 0.989 |
| Extended resection | 2.774 (0.577-13.350) | 0.203 |
| PpoFEV1% | 0.019 (0.003-0.114) | **<0.001** |
| FEV1/FVC | 0.001 (0.000-0.020) | **<0.001** |
| FVC% | 2.352 (0.383-14.450) | 0.356 |

OR, odds ratio; CI, confidence interval; ppoFEV1%, the percentage of predicted postoperative forced expiratory volume in one second; FEV1/FVC, the ratio of forced expiratory volume in one second to forced vital capacity; FVC%, the percentage of forced vital capacity.
